# PD-1和PD-L1在肺类癌中的表达情况和其临床意义

**DOI:** 10.3779/j.issn.1009-3419.2016.12.07

**Published:** 2016-12-20

**Authors:** 明彪 李, 嵩 徐, 海洋 范, 宏毅 张, 颖 李, 永文 李, 明辉 刘, 红雨 刘, 军 陈

**Affiliations:** 300052 天津，天津医科大学总医院肺部肿瘤外科，天津市肺癌研究所，天津市肺癌转移与肿瘤微环境实验室 Department of Lung Cancer Surgery, Tianjin Key Laboratory of Lung Cancer Metastasis and Tumor Microenvironment, Tianjin Lung Cancer Institute, Tianjin Medical University General Hospital, Tianjin 300052, China

**Keywords:** 肺肿瘤, 肺类癌, PD-1, PD-L1, Lung neoplasms, Pulmonary carcinoid, Programmed death-1 (PD-1), Programmed death ligand-1 (PD-L1)

## Abstract

**背景与目的:**

肺类癌(pulmonary carcinoid, PC)是一种发病率极低的肺部原发性恶性肿瘤，临床预后与其病理特征密切相关。本研究旨在检测分析PC组织中PD-1 (programmed death-1)和PD-L1(programmed death ligand-1)的表达情况及其与类癌患者临床病理生理特征的相关性。

**方法:**

应用免疫组织化学法检测了20例PC石蜡包埋组织标本中PD-1和PD-L1的蛋白表达情况；应用Hscore(HS)评分系统(0-300)对肿瘤组织中PD-L1和PD-1的表达进行了分析评价。

**结果:**

PD-1和PD-L1在PC组织中的阳性表达率分别为40%(8/20)和45%(9/20)。其中，在吸烟PC患者中，PD-1的阳性表达率为63.64%，明显高于不吸烟的患者(11.11%, *P* < 0.05)；PD-1与PD-L1的阳性表达与类癌患者的年龄、性别、病理类型、临床分期以及有无转移均无明显的相关性(*P* > 0.05)。

**结论:**

PD-1和PD-L1的表达在40%左右的PC患者中呈阳性。其中，吸烟类癌患者肿瘤组织中PD-1的阳性表达率明显高于不吸烟患者。这些结果提示PD-1和PD-L1的阳性表达可能与PC的发生、发展存在一定的相关性。

## Abstract (en)

**Background and objective:**

The incidence of pulmonary carcinoid (PC) is very rare in primary lung malignant tumors, and the prognosis of this disease is closely associated with its pathological features. In this study, the expressions of programmed death 1 (PD-1) and programmed death ligand-1 (PD-L1) in lung carcinoid cells were detected, and the correlation between the expression and corresponding clinical physiological and pathologic features was further analyzed.

**Methods:**

The expressions of PD-1 and PD-L1 in 20 cases of PC paraffin-embedded tissue specimens were detected through immunohistochemistry. The H-score of immunohistochemical staining (range, 0-300) was employed to evaluate the expression of PD-1 and PD-L1 in the tumor tissues.

**Results:**

In the 20 cases of patients with PC, 40% (8/20) showed positive expressions of PD-1, and 45% (9/20) showed positive expressions of PD-L1. Significantly higher expressions of PD-1 were observed in the smoking patients than in the nonsmoking patients (63.64% *vs* 11.11%, *P* < 0.05). Furthermore, no significant relationship was found between the expressions of PD-1 and PD-L1 and the clinical characteristics of the patients, such as age, gender, pathological type, clinical stage, and metastasis (*P* > 0.05).

**Conclusions:**

Approximately 40% of PC patients had positive expressions of PD-1or PD-L1. The positive expression rate of PD-1 in the smoking patients was significantly higher than that in the nonsmoking patients. These results suggest that the expressions of PD-1 and PD-L1 may be associated with the occurrence and development of PC.

肺类癌(pulmonary carcinoid, PC)起源于支气管肺黏膜的神经内分泌细胞(Kitchitsky细胞)，属于肺的神经内分泌肿瘤。其病理类型分为典型类癌(typical carcinoid, TC)和非典型类癌(atypical carcinoid, AC)，其判断标准为：TC < 2个有丝分裂/高倍视野，AC为2个-10个有丝分裂/高倍视野，且可伴有组织坏死和结构破坏。根据国际诊断的标准，PC的生物学特性虽不活跃，但仍具有侵袭和转移能力，常见胸外转移部位为肝、骨、脑和肾上腺^[1-4]^。根据2015版世界卫生组织肺肿瘤分类，将TC、AC与小细胞肺癌(small cell lung cancer, SCLC)、大细胞神经内分泌癌因为均具有神经内分泌功能而归属于神经内分泌肿瘤(neuroendocrine tumor, NET)^[5]^。在发病率方面，PC是极少见的恶性肿瘤^[3]^，在美国一项大规模流行病学调查^[6]^显示其年发病率约仅为十万分之0.3-1.35，而我国尚无明确发病率统计结果。支气管PC患者年龄分布较广(4岁-86岁)，TC与AC中位发病年龄在50岁左右，男女无差异；但在 < 30岁的患者中，AC不足10%^[7]^。肺部的NET无特异性的临床症状，甚至大多数患者不会出现临床症状。部分患者可表现为神经内分泌症状，临床多特征性表现为急性腹泻、肤色潮红、心悸、哮喘样症状和头痛^[1, 8]^。PC的诊断主要依据组织病理，治疗首选外科手术切除，一般早期患者术后预后较好，5年生存率可达90%，然而仍有部分患者死于远处转移等并发症^[3, 9]^。

肿瘤的免疫治疗是目前研究的主要热点之一。免疫检查位点PD-1与其配体PD-L1可以负性调控T细胞介导的肿瘤杀伤反应，从而使肿瘤细胞逃离免疫系统的监视，避免免疫细胞的杀伤，因此PD-1与PD-L1的靶点抑制剂可以克服抗肿瘤细胞的免疫逃离，从而达到消灭肿瘤细胞的作用^[10]^。现有的研究表明，PD-1与PD-L1在一些恶性肿瘤如黑色素瘤、肾癌以及肺癌中存在异常的表达^[11, 12]^，且PD-1与PD-L1的表达可能与此类恶性肿瘤的临床病理特征和不良预后相关^[13]^。目前，针对PD-1与PD-L1的靶点抑制剂，如Nivolumab、Atezolizumab、Pembrolizumab、Durvalumab等，已在黑色素瘤、肾癌、肺癌等恶性肿瘤的免疫治疗中取得了良好的效果^[14-17]^。本研究旨在探讨PC组织中PD-1与PD-L1的表达情况和与患者的临床病理生理特征的关系，为进一步应用针对PD-1和PD-L1的PC免疫治疗提供理论依据。

## 1 材料与方法

### 1.1 患者基本临床资料与标本采集

20例病理明确的PC患者的组织标本均来自于天津医科大学总医院肺部肿瘤外科2008年1月-2015年12月的手术病例，组织标本的应用均通过医院伦理委员会的批准和患者的同意。其中，14例患者行肺叶切除术，6例患者行亚肺叶切除术；17例患者行了系统性淋巴结清扫术，3例患者因肿瘤最大直径 < 1 cm，且为周围型而未行淋巴结采样。患者的临床资料和特征见表 1，每例患者的病理检测均经我院两位病理科医师会诊确诊。

**1:** 20例肺类癌患者的临床特征和PD-1/PDL1的表达情况 The clinical characteristics of 20 patients with PC and their expressions of PD-1/PD-L1

Item	*n*	Proportion (%)	PD-1				PD-L1		
			(+)	(-)	*P*		(+)	(-)	*P*
Gender					0.603				0.999
Male	15	75	7	8			7	8	
Female	5	25	1	4			2	3	
Age (yr)					0.999				0.370
> 48.5	10	50	4	6			3	7	
≤48.5	10	50	4	6			6	4	
Smoking					0.028				0.999
Yes	11	55	7	4			5	6	
No	9	45	1	8			4	5	
Histology					0.197				0.999
TC	9	45	2	7			4	5	
AC	11	55	6	5			5	6	
Lymph node metastasis					0.347				0.617
Yes	5	25	3	2			3	2	
No	15	75	5	10			6	9	
TNM stage					0.256				0.805
Ⅰ	11	55	3	8			6	5	
Ⅱ	1	5	1	0			0	1	
Ⅲ	8	40	4	4			3	5	
Ⅳ	0	0	0	0			0	0	
Stage					0.648				0.670
Early	12	60	4	8			6	6	
Advanced	8	40	4	4			3	5	
TC: typical carcinoid; AC: atypical carcinoid.									

### 1.2 免疫组化

组织标本的固定及包埋等处理过程按我院病理科常规，肿瘤组织标本应用4 μm的石蜡切片。首先应用原装脱蜡液中经过40 min脱洗进行脱蜡处理，再分别经无水酒精、95%酒精、85%酒精及70%酒精各10 min进行水化，在微波炉中使用相应的抗原修复液进行肿瘤组织的抗原修复30 min。

PD-1与CD8行双染法染色：CD8抗体来自基因科技(上海)公司C8/114B，T细胞鼠抗人单克隆抗体，经过1:25稀释后使用；PD-1抗体是来自SIGMA公司的兔抗人单克隆抗体，经1:50稀释后使用。PD-1附着后4 ℃下过夜，CD8使用迈新试剂的免疫组化双染试剂盒染色。PD-L1抗体是来自Cell Signaling Technology，(E1L3N)XP兔抗人单克隆抗体，经稀释液1:100稀释后使用，在4 ℃下过夜，使用中杉金桥BAD显色剂，三步显色。

PD-1是表达于CD8阳性T细胞的细胞膜，使用PD-1与CD8双染色，避免其他非CD8阳性T细胞的干扰。PD-1的染色强度与PD-L1相同，然而由于T细胞数目有限，按照惯例，使用PD-1阳性的细胞数目进行评分。

PD-L1是表达于肿瘤细胞及周围炎性细胞的胞膜，根据其染色强度和染色比例进行半定量数据分析。按染色强度分0、1+、2+和3+。0：在背景下没有细胞膜任何着色，记为阴性；1+：在细胞膜上轻度的染色，较2+、3+弱；2+：在细胞膜可见中等强度的染色；3+：在细胞膜可见强阳性，甚至胞膜周围出现深然颗粒。H-scores(HS)评分系统^[18]^：在400×的高倍镜视野下HS=阳性细胞比例×染色强度，分别随机取5个-8个视野并计算HS平均值。

### 1.3 统计学分析

此次研究的主要目的是探索在PC中PD-1/PD-L1的表达情况和其与肺癌患者的不同病理分类、病理分期及临床特征的关系。数据分析应用IBM公司的SPSS软件(版本21)，采用卡方检验或*Fish*精确检验法，分别计算PD-1和PD-L1阳性患者的临床特征及病理类型的关系。*P* < 0.05为差异具有统计学意义。

## 2 结果

### 2.1 患者资料与临床特征

患者的临床特征如表 1所示：在20例PC患者中，男性15例，女性5例；年龄范围是14岁-71岁，中位年龄为48.5岁；11例有吸烟史，9例无吸烟史；20例患者均接受了手术治疗，具有明确的病理诊断，其中，TC 8例，AC 12例；伴有淋巴结转移者5例(均为AC)，侵及上腔静脉大血管者3例；临床病理分期，Ⅰ期-Ⅱ期12例，Ⅲ期8例。

### 2.2 PD-1在PC组织中CD8^+^淋巴细胞上的表达情况

PD-1表达于肿瘤细胞周围浸润的CD8(+)的T淋巴细胞的胞膜。如图 1A-图 1D所示，应用PD-1/CD8双染的方法来确定PD-1的阳性表达情况，在显微镜下，PD-1为深蓝色，CD8为浅红色，二者均表达在淋巴细胞的胞膜上，部分抗体可呈现颗粒状聚集，二者叠加在镜下显示为蓝黑色。在所有类癌患者的肿瘤组织中，PD-1(+)和CD8(+)双阳性的T淋巴细胞数均大于50，其中部分PC组织中可见只表达CD8(+)PD-1(-)的T淋巴细胞，但未见PD-1(+)CD8(-)的淋巴细胞。如表 1所示，20例类癌患者中PD-1表达阳性者8例，阳性表达率为40%(8/20)。在吸烟患者中PD-1的阳性表达率为63.64%(7/11)，明显高于不吸烟患者(11.11%, 1/9)(*P*=0.028)；PD-1的阳性表达与患者的性别、年龄、病理类型、病理分期和有无转移无明显相关性(*P* > 0.05)。

**1:**
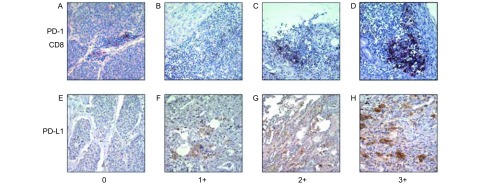
PD-1和PD-L1在肺类癌组织中的表达(×400)。A-D：PD-1和CD8双染色。A：PD-1染色阴性，单纯CD8染色，呈现淡红的；B：PD-1染色1+，在细胞膜上轻度的蓝染色；C：PD-1染色2+，在细胞膜可见中等强度的深蓝染色；D：PD-1染色3+, 在细胞膜可见蓝黑染色强阳性，甚至胞膜周围出现深然蓝黑颗粒。E-H：PD-L1染色。E：PD-L1染色0，在背景下没有细胞膜任何着色，即表达阴性；F：PD-L1染色1+，在细胞膜上轻度棕红色的染色，较2+、3+弱；G：PD-L1染色2+，在细胞膜可见中等强度棕色的染色；H：PD-L1染色3+，在细胞膜可见深棕色强阳性，甚至胞膜周围出现深然颗粒。染色强度分0、1+、2+和3+。 The expression of PD-1 and PD-L1 in pulmonary carcinoid tissues by Immunohistochemistry detection (Original Magnification, ×400). A-D: The staining for PD-1 and CD8. A: PD-1 staining was negative, pure CD8 staining, showing pink; B: PD-1 stained 1+, mild blue staining on the cell membrane; C: PD-1 staining 2+, PD-1 visible blue staining in the cell membrane of moderate intensity, pure CD8 staining, showing light red; D: PD-1 staining 3+, PD-1 Black and blue visible in the cell membrane staining strongly positive, then even the membrane around the deep blue-black particles. E-H: The staining for PD-L1. E: PD-L1 staining 0, in the context of the membrane without any coloring, i.e. negative expression; F: PD-L1 staining 1+, slightly brown-red in the cell membrane staining, representing 2+, 3+ weak: G: PD-L1 staining 2+ in the cell membrane visible moderate brown staining; H: PD-L1 staining 3+, visible in dark brown membrane strongly positive, even around the deep natural membrane particles. Staining intensity points: 0, 1+, 2+, and 3+.

### 2.3 PD-L1在PC细胞中的表达情况

如图 1E-图 1H所示，PD-L1表达于肿瘤细胞的胞膜上，DAB显色后镜下为棕色。当HS > 0时，认为PD-L1有表达的标准评判，20例类癌中有9例患者PD-L1表达为阳性，其阳性表达率为45%(9/20)。与PD-1的表达不同的是，在吸烟的患者中PD-L1的阳性表达率为45.45%(5/11)，与不吸烟的患者基本相似(44.44%, 4/9)，组间差异无统计学意义(*P* > 0.05)；此外，PD-L1的阳性表达与患者的性别、年龄、病理类型、病理分期和有无转移均无明显相关性(*P* > 0.05)(表 1)。

表 2所示为PD-1/PD-L1表达阳性的PC患者的临床具体特征，其中在PD-1或/和PD-L1表达阳性的患者中，5例患者同时存在PD-1和PD-L1的表达阳性，共同阳性率为25%(5/20)。这5例患者的临床特征如下：有吸烟史、男性4例，无吸烟史、女性1例；患者年龄在25岁-70岁之间，中位年龄48岁；TC 1例，AC 4例。由于临床病例数的不足，不能分析出此类患者的PD-1和PD-L1的两者阳性表达是否存在相关性。

**2:** 肺类癌PD-1/PD-L1阳性患者的临床特征 Clinical features of PC patients with the positive expressions of PD-1/PD-L1

Patient	Sex	Age (yr)	Smoking (yr)	Histology	Position	Diameter (cm)	N disease	Stage	TNM stage	PD-1(+) CN	PD-L1 Hscore	Status
9	Male	49	35	AC	Peripheral	2.2	Unkonwn	Ⅰa	T1bN0M0	50	0	Survive
1	Male	40	20	AC	Central	5	Positive	Ⅲa	T4N0M0	> 200	0	Survive
14	Male	66	50	TC	Central	4.5	Positive	Ⅱa	T2bN0M0	> 200	0	Survive
12	Male	46	10	TC	Central	2.2	Negative	Ⅰa	T1cN0M0	100	10	Survive
5	Male	70	40	AC	Peripheral	2.2	Positive	Ⅲa	T1cN2M0	100	80	Survive
6	Male	49	20	AC	Central	5	Positive	Ⅲb	T4N2M0	100	120	Death
16	Male	48	30	AC	Central	1.2	Negative	Ⅰa	T1bN0M0	100	120	Survive
11	Female	25	0	AC	Central	1.8	Positive	Ⅲa	T1bN2M0	40	30	Survive
13	Male	25	0	AC	Central	2	Negative	Ⅰa	T1bN0M0	0	40	Survive
17	Female	28	0	TC	Central	2.5	Negative	Ⅰa	T1cN0M0	0	40	Survive
19	Male	57	20	TC	Peripheral	1	Negative	Ⅰa	T1aN0M0	0	40	Survive
3	Male	47	0	TC	Central	1	Negative	Ⅰa	T1aN0M0	0	80	Survive
CN: cell number.												

### 2.4 PD-1与PD-L1阳性表达与患者预后的关系

20例PC患者术后随访时间为15个月-96个月，平均随访时间为48个月，其中1例晚期AC患者因术后7个月出现脑转移而死亡，其余19例患者现均生存，故目前尚不能分析PD-1/PD-L1的阳性表达与患者的预后相关性。如图 2A-图 2D所示，死亡患者为男性，49岁，胸部电子计算机断层扫描显示右肺上叶肿物伴右肺中叶转移和纵隔淋巴结肿大，行右肺中上叶切除，系统淋巴结清扫术，术后病理诊断为右上肺AC伴右肺中叶肺内转移和纵隔淋巴结的转移(T4N2M0, Ⅲb)，患者术后接受标准的TP方案(多西他赛130 mg+奥沙利铂220 mg)化疗4次，7个月后因脑转移死亡。PD-1/PD-L1的免疫组化结果如图 2E-图 2H所示，在400倍镜下，PD-1阳性细胞数大于100(图 2E-图 2F)，PD-L1的HS大于120(图 2G-图 2H)，为PD-1和PD-L1双阳性的患者。

**2:**
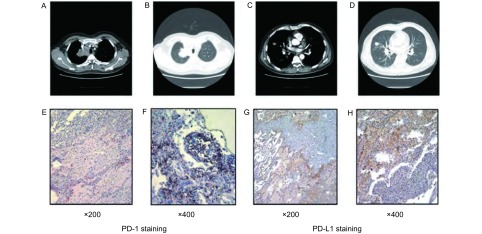
肺类癌患者的胸部CT和其肺癌组织中PD-1/PD-L1表达情况。A-B：右肺上叶见一大小为4 cm×5 cm肿物，侵及上腔静脉；C-D：右肺中叶亦见一大小为2 cm×2 cm肿物；E-F：PD-1表达呈强阳性(200×/400×)；G-H：PD-L1表达呈强阳性(200×/400×)。 The chest CT scan and the expression of PD-L1 and PD-1 for the patient who died due to pulmonary carcinoid. A-B: There was a tumor size of 4 cm×5 cm mass in the right upper lobe, which invaded the superior vena cava; C-D: There was also a tumor size of 2 cm×2 cm lesion in the right middle lobe; E-F: the strongly positive expression of PD-1 (3+; 200×/400×); G-H: the strongly positive expression of PD-L1 (3+; 200×/400×).

## 3 讨论

PC在肺癌中发病率极低，确诊以病理诊断为主。其特异性免疫组化标记物主要有chromogranin A (CgA)、CD56、synaptophysin、neuron-specific enolase (NSE)，然而这些标记物与PC的临床特征及预后无明确相关性。PC患者术后预后一般较好，然而部分PC亦有高的侵袭性，易导致患者死亡^[9]^。目前，肿瘤的免疫治疗是研究的主要热点，其中PD-1或PD-L1的阻断剂在包括肺癌在内的恶性肿瘤的初步临床研究中取得可靠的治疗效果^[10]^。虽然，PD-1或/和PD-L1的表达尚不能完全确定为PD-1或PD-L1的阻断剂治疗有效与否的主要标记物，但在许多的研究^[19]^中PD-L1的表达仍被建议作为一个使用抗PD-1/PD-L1疗法的标记。目前，PC组织中的PD-1/PD-L1表达及抗PD-1/PD-L1治疗的研究报道甚少。

2014年底美国食品和药物管理局标准规定，肺癌组织中PD-L1的表达被建议作为一个使用抗PD-1/PD-L1疗法标记。本项研究发现PC中PD-1/PD-L1表达率分别为40%和45%，完全符合NSCLC的表达率，文献^[20-22]^报道PD-1/PD-L1阳性率一般分别为40%-60%与35%-95%，特别是在吸烟史的类癌患者中，PD-1的阳性表达率为63.64%，明显高于不吸烟的患者。因此对于晚期肺类癌，使用抗PD-1/PD-L1的疗法提供了一种新的治疗可能。已经有研究^[23]^表明PD-1抗体的效果与肿瘤细胞的突变程度有关系，突变越多效果可能就越好。如恶性黑色素瘤的突变频率很高，所以这种肿瘤对PD-1抗体治疗很敏感^[24]^。研究报道在458例有明确吸烟史的患者中，使用Opdivo(PD-1抑制剂)的效果明显优于无吸烟史患者(HR=0.7)；而对于从来不抽烟的118例患者，从HR值来看，Opdivo效果和多西他赛无明显差异。研究^[15]^认为抽烟会让肿瘤细胞有更多的突变。已经有研究表明PD-1抗体的效果跟肿瘤细胞的突变程度有关系，突变越多效果可能就越好。恶性黑色素瘤的突变频率很高，所以这种肿瘤对PD-1抗体治疗很敏感^[24]^。在20例患者随访过程中，目前只有1例患者死于肿瘤的转移，该患者的PD-1/PD-L1的表达率均为强阳性。文献[9]报道PC的死亡率极低，与本组患者的目前随访结果相符。然而在一些恶性肿瘤如黑色素瘤和肾癌的研究中，亦有文献[11, 25]报道PD-1/PD-L1高表达的患者提示预后不良。由于本组患者总体例数和死亡患者的比例均较低的原因，在该研究中不能证实这一点。

目前，由于不同生产厂家的PD-1/PD-L1抗体的效价不同，免疫组化染色的半定量统计的标准尚未统一和PD-1/PD-L1阳性临界值公认标准的缺乏，使得不同文献报道的肺癌患者中PD-1/PD-L1的阳性率差别很大。在本组实验中，我们参用了大多文献常用的计量标准即PD-1/CD8双阳性细胞计数大于零为阳性，PD-L1的表达以HS > 0为阳性的标准进行了判断。从而在本组类癌患者中，PD-1和PD-L1的阳性表达率均大于40%，但这些患者能否在针对PD-1和PD-L1抗体的治疗中获益目前仍缺乏必要的临床研究。大宗此类病例的进一步研究必将为PD-1/PD-L抗体在临床的应用奠定基础。

综上所述，此次研究中，PC组织中的PD-1与PD-L1的表达率明显高于远癌肺组织，应用PD-1/PD-L抗体的免疫治疗也许可以在PC患者的治疗中获益。
